# Integrating Phenotypic and Genomic Data with Machine Learning to Predict Antimicrobial Resistance and Identify Genetic Biomarkers in *E. coli*

**DOI:** 10.3390/ijerph23050561

**Published:** 2026-04-27

**Authors:** Sarah Halleluyah Adeyemi, Roshan Paudel

**Affiliations:** Bioinformatics Program, Department of Computer Science, School of Computer Science, Mathematics and Natural Sciences, Morgan State University, Baltimore, MD 21251, USA; saade13@morgan.edu

**Keywords:** *Escherichia coli*, antimicrobial resistance, phenotypic data, machine learning, phylogenetic analysis, genomic biomarkers

## Abstract

**Highlights:**

**Public health relevance—How does this work relate to a public health issue?**

- Antimicrobial Resistance (AMR) in *Escherichia coli* (*E. coli*) is an increasing global public health problem that has resulted in treatment failures, increased healthcare costs, and mortality rates.
- This study combines phenotypic data of antimicrobial susceptibility testing and genomic analysis through machine learning to better predict biologically relevant patterns in antimicrobial resistance.

**Public health significance—Why is this work of significance to public health?**

- This study shows that machine learning models like XGBoost and Random Forest are effective at classifying antimicrobial resistance in large clinical datasets.
- Identification of key resistance biomarkers (e.g., gyrA, parC, blaCTX-M-15) is associated with a better understanding of the genetic mechanisms of antimicrobial resistance.

**Public health implications—What are the key implications or messages for practitioners, policy makers, and/or researchers in public health?**

- Data-driven prediction models may be helpful in antimicrobial stewardship programs by facilitating earlier detection of resistant pathogens.
- Combining machine learning with genomic surveillance can improve global antimicrobial resistance monitoring systems and inform evidence-based antibiotic use.

**Abstract:**

Antimicrobial resistance in *Escherichia coli (E. coli*) is a major public health concern globally, driven by increased resistance to commonly used antimicrobial agents such as β-lactams and fluoroquinolones. The main goal of our research is to develop a machine learning framework to predict antimicrobial resistance in *E. coli* by integrating antimicrobial susceptibility testing data with genomic biomarker analysis. A dataset comprising 17,122 *E. coli* clinical isolates was obtained from the Bacterial and Viral Bioinformatics Resource Center (BV-BRC). After preprocessing, fivefold cross-validation was used to train and test five machine learning models: Random Forest, XGBoost, Support Vector Machine, Logistic Regression, and k-Nearest Neighbors. The highest-performing model was XGBoost, with 0.86 accuracy and 0.932 ROC-AUC, followed by Random Forest, with 0.82 accuracy and 0.89 ROC-AUC. Phylogenetic analysis revealed that resistant isolates clustered together relative to the reference genome of *E. coli* K-12 MG1655. Genomic biomarkers such as gyrA, parC, CTX-M-15, OXA-1, and various multidrug efflux pumps were identified by the Comprehensive Antibiotic Resistance Database (CARD) and ResFinder as significant resistance determinants in this study. In conclusion, this study demonstrates that combining antimicrobial susceptibility testing with machine learning and genomic biomarkers is a powerful framework for predicting antimicrobial resistance in *E. coli*.

## 1. Introduction

Antimicrobial resistance (AMR) is one of the major global health challenges of the 21st century, affecting the efficacy of many commonly used antibiotics and increasing morbidity, mortality, and healthcare costs worldwide. According to a global study published in The Lancet, bacterial AMR was estimated to have directly caused 1.27 million deaths, while 4.95 million deaths were associated with AMR in 2019 globally [1]. This makes AMR a leading cause of mortality, on par with major diseases such as HIV and malaria. Beyond reducing survival rates, AMR also leads to significant healthcare costs due to extended hospital stays, additional testing, and the use of more expensive or toxic drugs [2]. *E. coli* is a major cause of community-acquired and healthcare-associated infections. It has become increasingly resistant to several classes of antibiotics, posing a serious problem for clinicians and healthcare systems [3,4]. Early and accurate detection of resistance in *E. coli* is therefore crucial to enhance patient safety, inform empirical treatment, and prevent the spread of resistant strains [4]. Conventional antimicrobial susceptibility testing, or AST, is considered the gold standard; however, it requires considerable resources and time. Whole-genome sequencing (WGS) technology now enables the detailed sequencing of bacterial genomes, allowing for the development of models that predict resistance directly from the bacterial genome [5,6].

However, the complexity and volume of genomic data require advanced computational methods to extract meaningful clinical insights. While stewardship programs are currently in progress and an understanding of antibiotic risks is increasing [7,8,9], a disconnect remains between pathogen resistance and prescribed drugs. An example of a problem increasingly under public scrutiny is ciprofloxacin, a fluoroquinolone antibiotic that has been widely prescribed since the early 2000s and is listed among the WHO’s Essential Medicines [8]. It is effective against various strains of both Gram-negative and Gram-positive bacteria, including those responsible for urinary tract, respiratory, bone/joint, and gastrointestinal infections [9,10]. However, its widespread use has led to the emergence of resistance globally [11,12,13,14]. Although restrictions on antibiotic use can help reduce selective pressure [15], resistance often persists due to the fitness benefits of resistant clones and horizontal gene transfer [12,13].

In recent years, machine learning (ML) has been recognized as one of the most impactful tools for predicting antimicrobial resistance (AMR). Compared to traditional statistical methods, ML algorithms such as Random Forests, Support Vector Machines (SVMs), Logistic Regression, and XGBoost can capture complex, nonlinear relationships in large datasets. These models provide fast, reliable predictions that can help clinicians select the most effective treatments and support antimicrobial stewardship programs [16]. While previous studies mainly focus on whole-genome sequencing (WGS) data, this research advances by using ML to analyze large-scale phenotypic antimicrobial susceptibility testing (AST) data, with phylogenetic analysis and biomarker discovery as secondary methods. This combined approach enhances prediction accuracy and bioinformatic interpretability, making it particularly valuable in hospital settings where genomic sequencing resources may be limited. For example, Mintz used hospital-based clinical data to predict ciprofloxacin resistance using logistic and gradient-boosting models but did not incorporate phenotypic or phylogenetic data [16]. Similarly, public AMR prediction challenges, such as the Kaggle “AMR Benchmark Dataset,” focus on whole-genome predictors without linking results to phenotypic AST outcomes. In contrast, this study bridges that gap by integrating large-scale phenotypic AST data with machine learning and genomic biomarker screening to improve resistance classification and interpretability. By combining both data types, this research offers a more clinically applicable framework for early AMR detection and genomic insights.

Although machine learning and deep learning approaches have been widely applied in antimicrobial resistance prediction, many existing studies rely primarily on genomic data and complex models that are not easily applicable in routine clinical settings. The implementation of these approaches is usually associated with difficulties in scalability, data availability, and real-time processing. Moreover, the limited use of phenotypic antimicrobial susceptibility data limits their direct clinical application. Thus, models that are both accurate and practical (using routinely available data while maintaining biological interpretability) are still required.

Recent studies have explored the use of machine learning for antimicrobial resistance prediction using both genomic and phenotypic data. For example, Nguyen et al. [17] used machine learning models to forecast AMR using genomic features and showed that it is possible to predict this disease accurately, but these models might not be applied in the real clinical setting. Likewise, Drouin et al. [18] presented a resistance-prediction approach using genomic data, but it faced scalability and interpretability challenges. The same phenotypic data were used in other studies, such as those by Yang et al. [19], but with smaller datasets and decreased generalizability. Also, benchmarking initiatives such as the AMR prediction challenges [20] did not emphasize the importance of fully integrating phenotypic outcomes, even though they relied primarily on genomic predictors.

Conversely, this paper utilizes big data of phenotypic antimicrobial susceptibility testing (AST) and machine-learned and genomic biomarker validation. The methodology focuses on clinical applicability (based on routinely available data) and improved interpretability (biological validation of resistance mechanisms).

Based on recent developments and identified shortcomings, this study presents an integrated framework for machine learning and bioinformatics to predict antimicrobial resistance in *E. coli*. For this purpose, a dataset of clinical isolates was collected from the Bacterial and Viral Bioinformatics Resource Center (BV-BRC), and several machine learning algorithms were evaluated for predicting antimicrobial resistance phenotypes based on phenotypic susceptibility data. In addition, genomic biomarker screening and phylogenetic analysis were conducted to predict resistance-associated genetic determinants. The major objective of this study is to propose an effective framework for predicting antimicrobial resistance and to improve data-driven approaches for its prediction. Unlike previous research, which relies primarily on genomic sequencing data, this research combines large-scale phenotypic antimicrobial susceptibility testing with machine learning and genomic biomarker analysis, offering a clinically applicable, resource-efficient framework for predicting antimicrobial resistance.

## 2. Materials and Methods

**Figure 1:**
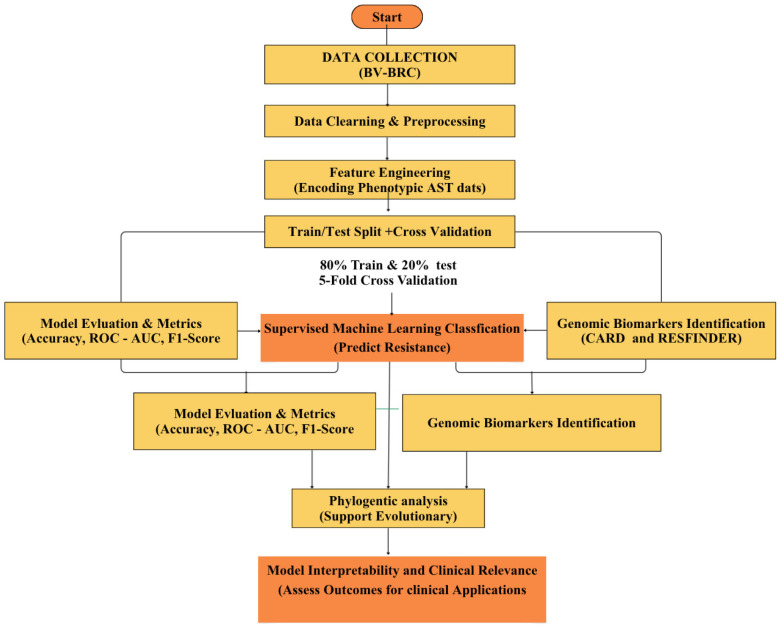
Analytical Workflow for Machine Learning-Based Classification and Biomarker Identification in *E. coli.* This figure represents supervised machine learning to predict antimicrobial resistance with phenotypic AST data, further performed before biological interpretation by genomic biomarker validation (CARD and ResFinder) and phylogenetic analysis.

The analytical workflow of the study is shown in Figure 1 below.

### 2.1. Datasets

The dataset used in this study was obtained from the Bacterial and Viral Bioinformatics Resource Center (BV-BRC), a comprehensive bioinformatics repository maintained by the National Institute of Allergy and Infectious Diseases (NIAID). It included 17,122 *E. coli* clinical isolates collected from various clinical and geographical sources, tested against multiple antibiotics across major drug classes, including β-lactams, fluoroquinolones, carbapenems, aminoglycosides, macrolides, and sulfonamides. Each isolate was annotated with antibiotic susceptibility results according to the Clinical and Laboratory Standards Institute (CLSI) or the European Committee on Antimicrobial Susceptibility Testing (EUCAST) guidelines. Data fields included isolate ID, antibiotic name, testing method (MIC or disk diffusion), testing standard, and phenotypic classification (resistant, intermediate, or susceptible). After preprocessing, 16,740 isolates remained for analysis. The dataset showed moderate class imbalance, with resistant isolates accounting for approximately 68% of the total entries and susceptible isolates accounting for 32%. Antibiotics such as ampicillin, ciprofloxacin, and ceftriaxone exhibited the highest resistance rates, while ertapenem and meropenem had lower resistance frequencies.

### 2.2. Data Cleaning and Preprocessing

To ensure analytical consistency, the raw phenotypic AST data were cleaned. Each observation is a pairing of isolates and antibiotics and not a single isolate. The prediction of models, hence, is the susceptibility to particular antibiotics. Excluded were isolates with unclear or missing antibiotic results. Using the antibiotic name and the genome ID, duplicate entries were eliminated. To improve class clarity, intermediate isolates were removed from the machine learning pipeline to reduce classification ambiguity; however, this may limit real-world applicability where intermediate resistance is clinically relevant, and phenotypic labels were standardized to ‘Resistant’ (R = 1) and ‘Susceptible’ (S = 0). LabelEncoder was used for the variables, and min-max normalization was applied to scale the final feature matrix to 0–1, ensuring consistent feature weighting. The phenotypic antimicrobial susceptibility data were used to derive the input features. The names of the antibiotics were coded into categorical variables, and the results of susceptibility were referred to as resistant or susceptible. Database information on genomics was not utilized as input features but rather used in downstream validation of biomarkers. To preserve class distribution, stratified sampling was used to divide the cleaned dataset into training (80%) and testing (20%) sets.

### 2.3. Machine Learning Algorithms

To evaluate predictive performance across models, five supervised algorithms were applied to assess their performance in antimicrobial resistance prediction from phenotypic data. These included Random Forest and XGBoost, which are ensemble-based [21,22], and Support Vector Machine and Logistic Regression, which are linear models and considered basic models [23,24,25,26,27,28,29]. The distance-based classification method, k-Nearest Neighbors, was incorporated in comparison.

Tree-based models were selected for their ability to capture nonlinear interactions and complex feature relationships, which are common in biological dataset [30]. Linear and distance-based models were included as baselines and to evaluate model generalization across the dataset. All models were trained and tested under identical conditions to ensure consistency.

### 2.4. Validation and Training of Models

The models were trained on 80% of the dataset and tested on the remaining 20% with stratified sampling. Model performance was measured using 5-fold cross-validation to ensure robustness and low overfitting. Hyperparameters were optimized using grid search, where applicable.

### 2.5. Performance Evaluation

Model performance was evaluated using several metrics, including accuracy, precision, recall, F1-score, and the area under the receiver operating characteristic (ROC) curve (ROC-AUC). These metrics have been chosen in order to obtain an extensive evaluation of the performance of the models, especially in the case of class imbalance. ROC-AUC was then used to determine the ability of the models to discriminate between resistant and susceptible isolates at different thresholds.

- Accuracy (Acc) = (TP + TN)/(TP + TN + FP + FN).
- Precision (P) = TP/(TP + FP).
- Recall (R) = “TP/(TP + FN)”.
- F1-score (F1) = “2 × (P × R)/(P + R)”.
- Area Under the Receiver Operating Characteristic Curve “(ROC–AUC)”.

The confusion matrices and ROC–AUC curves were plotted for each model using the RocCurveDisplay function in scikit-learn. These visualizations were used to compare the sensitivity and specificity of models across multiple antibiotic classes.

### 2.6. Visualization and Feature Interpretation

To assess model interpretability, feature-importance plots were created for Random Forest and XGBoost models. Key features related to antibiotic types and resistance classes were identified to determine which variables most affected the prediction results. Visualization tools, such as “Matplotlib (version 3.8) and Seaborn (version 0.13)”, were used to generate confusion matrices, ROC curves, and correlation heatmaps.

### 2.7. Phylogenetic Analysis

Whole-genome FASTA sequences of *E. coli* clinical isolates were obtained from the BV-BRC database. For evolutionary analysis, the genome of *E. coli* K-12 MG1655 (GenBank accession U00096.3) was also retrieved from NCBI. The sequences were aligned using MAFFT (Multiple Alignment using Fast Fourier Transform), which operates in auto mode to optimize parameters based on the dataset size and complexity. The aligned sequences were used with FastTree to generate a maximum-likelihood phylogenetic tree under the General Time Reversible (GTR) substitution model. Using Archaeopteryx JS, the tree was visualized with color-coded annotations, allowing comparison of the ingroup (*E. coli* strains), the reference sequence (K-12), and the outgroups (closely related Enterobacteriaceae species).

### 2.8. Identification of Genomic Biomarkers

Genomic biomarkers were identified through a comparative and literature-guided bioinformatics approach. Due to the limited availability of paired resistant and susceptible genome sequences, this study employed an in silico comparison against the *E. coli* K-12 reference and cross-referenced known resistance loci reported in the literature and in AMR databases. Genes with established links to resistance, including gyrA, gyrB, parC, parE, acrA, acrB, mdtK, blaTEM, and blaCTX-M, were examined. Multiple sequence alignment using MAFFT identified key substitution positions, such as S83L, D87N, and S80I, which are associated with fluoroquinolone resistance. These biomarkers were validated using the Comprehensive Antibiotic Resistance Database (CARD) and ResFinder.

## 3. Results

### 3.1. The Distribution of Antibiotics Testing and Resistance Patterns

**Figure 2:**
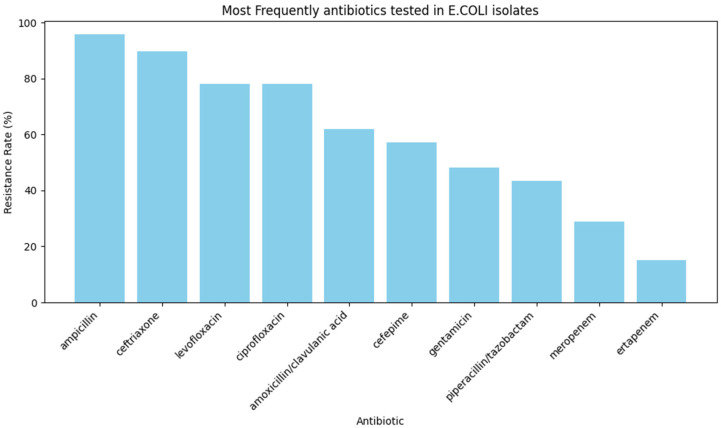
Top 10 most frequently tested antibiotics in *E. coli* isolates. It displays the proportion of antimicrobial susceptibility tests conducted for each antibiotic in the BV-BRC dataset. Ampicillin, ceftriaxone, levofloxacin, and ciprofloxacin were the most frequently evaluated, highlighting their widespread clinical use. In contrast, carbapenems such as meropenem and ertapenem were less often tested, reflecting their role as last-resort treatments for multidrug-resistant infections.

The dataset included a wide variety of antibiotics, but a subset stood out as the most frequently tested. These included ampicillin, ceftriaxone, ciprofloxacin, levofloxacin, gentamicin, amoxicillin/clavulanic acid, cefepime, piperacillin/tazobactam, meropenem, and ertapenem (Figure 2). These antibiotics represent both commonly prescribed medications and critical last-resort treatments. For example, ampicillin and ciprofloxacin are frequently used in clinical practice, whereas carbapenems, such as meropenem and ertapenem, are reserved for severe or multidrug-resistant infections.

### 3.2. Machine Learning Model Performance

The machine learning models, phylogenetic analysis, and genomic biomarker identification are presented below. Five ML algorithms (Random Forest, eXtreme Gradient Boosting, Support Vector Machine, K-Nearest Neighbors, and logistic regression) were used to classify antimicrobial resistance in *E. coli* based on phenotypic antimicrobial susceptibility testing (AST) data. The models were evaluated using metrics such as accuracy, precision, recall, F1-score, and ROC-AUC to assess their predictive performance.

The performance of all five machine learning models is summarized in Table 1. XGBoost and Random Forest achieved the highest predictive accuracy among the models tested.

**Figure 3:**
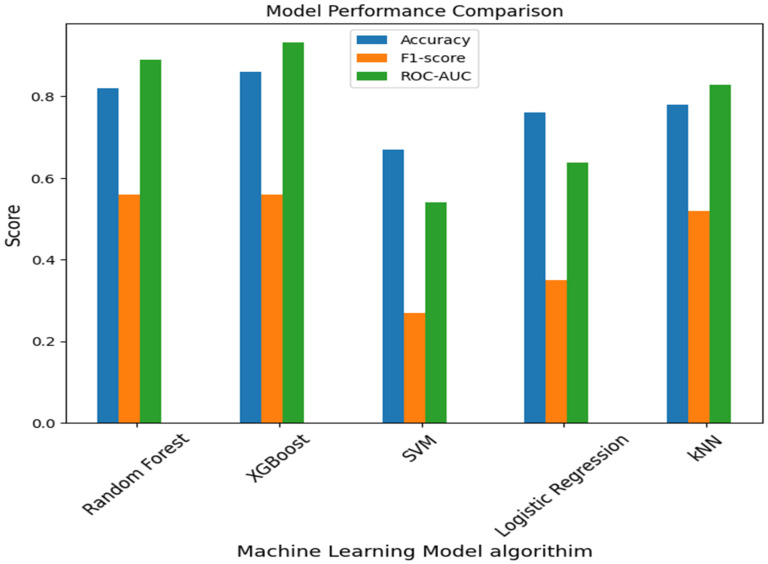
A comparative performance of five machine learning models. The model performance metrics, including accuracy, F1-score, and ROC-AUC, are shown for the five machine learning algorithms used. XGBoost achieved the highest predictive accuracy (0.86) and area under the curve (0.932), followed by Random Forest, demonstrating the strength of ensemble methods in AMR prediction.

Tree-based ensemble methods outperformed all other approaches. XGBoost achieved the highest performance (accuracy = 0.86, ROC-AUC = 0.932), followed closely by Random Forest (accuracy = 0.82, ROC-AUC = 0.89). Both models achieved strong classification performance for susceptible and resistant isolates but struggled to classify the minority class (“Intermediate”), which comprised fewer than 25 samples. In contrast, SVM and logistic regression performed poorly, with ROC-AUC values of 0.54 and 0.64, respectively. These models exhibited a strong majority-class bias (susceptible) and showed little to no ability to accurately identify resistant or intermediate isolates. The kNN model also demonstrated good predictive performance (accuracy 0.78, ROC-AUC 0.828), but its precision and recall suggest potential overfitting to the majority class. SVM and LR achieved lower accuracies of 0.67 and 0.76, respectively, suggesting that linear models are less effective at handling the complex, nonlinear feature relationships in phenotypic AMR data. Ensemble-based approaches (XGBoost and Random Forest) provided the best trade-off between bias and variance, making them robust models for antimicrobial resistance classification. See Figure 3 below for the visualization of the performance accuracy.

### 3.3. The Resistance Distribution Across Antibiotics

**Figure 4:**
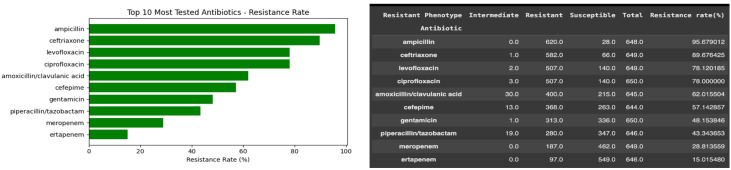
Resistance rate of the top 10 most frequently tested antibiotics. Fluoroquinolones (ciprofloxacin, levofloxacin) and β-lactams (ampicillin, ceftriaxone) showed the highest resistance levels, whereas carbapenems retained substantial activity.

The antibiotic resistance distribution derived from the cleaned BV-BRC dataset among the tested antibiotics includes ampicillin, ciprofloxacin, ceftriaxone, and cefotaxime. These antibiotics demonstrated the highest resistance rates, while imipenem and meropenem maintained significant efficacy. This distribution aligns with WHO’s global AMR surveillance reports, which identify fluoroquinolones and β-lactams as the most compromised antibiotic classes due to decades of overuse in clinical and agricultural settings. The proportion of resistant isolates was calculated for each antibiotic. The results showed that ampicillin had the highest resistance rate (>95%), followed by third-generation cephalosporins such as ceftriaxone and cefotaxime, which also exhibited extremely high resistance (Figure 4). Fluoroquinolones (ciprofloxacin and levofloxacin) demonstrated resistance rates of 70–80%, indicating reduced clinical utility, although some isolates remained susceptible. The differences were found descriptively but not formally tested statistically, and it should be taken into account in other studies.

**Figure 5:**
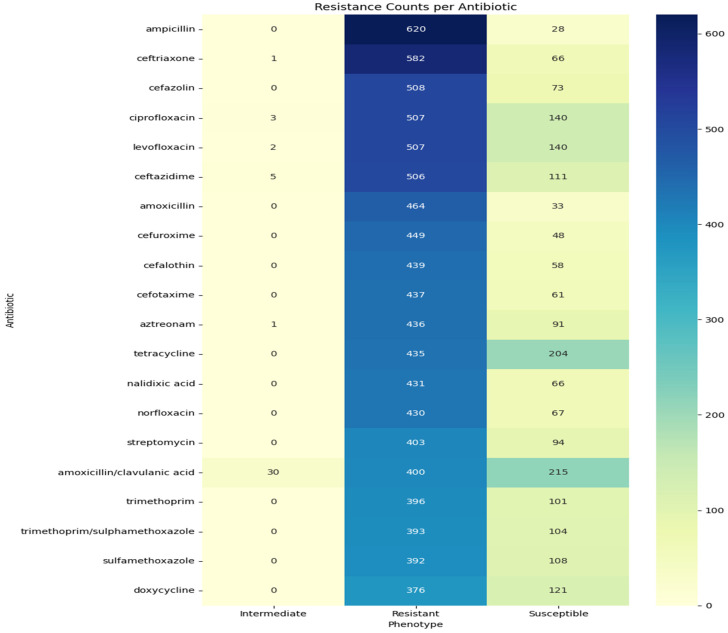
A Heatmap of Resistance per Count showing the resistance, susceptible, and intermediate isolate counts for the top 20 antibiotics. The heatmap helps visualize the proportions of resistance phenotypes within individual antibiotic classes. The darker blue colors indicate an increase in the number of resistant isolates, especially to ampicillin, ceftriaxone, and ciprofloxacin. Carbapenems and aminoglycosides were less dark, indicating a relatively low prevalence of resistance.

A heatmap was created showing the counts of resistant, susceptible, and intermediate isolates for the top 20 antibiotics (Figure 5). Ampicillin, ceftriaxone, and cefotaxime had the highest resistance counts, with almost no susceptible isolates. Ciprofloxacin and levofloxacin were mostly resistant, although susceptible isolates were still present. Aminoglycosides, such as gentamicin and amikacin, displayed a more balanced distribution. Carbapenems, especially meropenem, were generally susceptible; however, the presence of resistant isolates indicates a potential decline in their clinical effectiveness.

**Figure 6:**
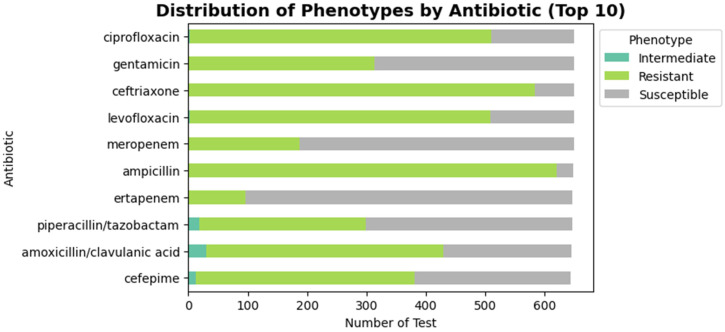
The distribution of resistant, susceptible, and intermediate phenotypes for the top 10 antibiotics. This figure demonstrates phenotypic heterogeneity observed between the antibiotics used in model training. The most predominant resistant phenotypes were ciprofloxacin, ampicillin, levofloxacin, and ceftriaxone, whilst higher susceptibility was observed with carbapenems, including meropenem and ertapenem, highlighting the disparity in selection pressure among antimicrobial groups.

A stacked bar chart was used to display the distributions of phenotypes for the top 10 most frequently tested antibiotics (Figure 6). Ampicillin was almost entirely resistant, confirming its ineffectiveness. Cephalosporins such as ceftriaxone and cefepime also displayed high resistance. By contrast, gentamicin and cefepime retained some effectiveness, with significant numbers of susceptible isolates. Carbapenems (meropenem, ertapenem) were largely susceptible, demonstrating their importance as reserve drugs, though resistant isolates were present in smaller numbers.

### 3.4. Phylogenetic Analysis

The phylogenetic tree showed distinct clustering patterns of the analyzed *E. coli* isolates. Ciprofloxacin resistant isolates usually clustered into different clades apart from the majority of the weak strains, indicating some evolutionary branches in relation to antimicrobial resistance. A few susceptible isolates were more tightly clustered with the reference strain *E. coli* K-12 MG1655, but resistant isolates were more separated in their branches, possibly reflecting the accumulation of resistance-related genetic changes.

**Figure 7:**
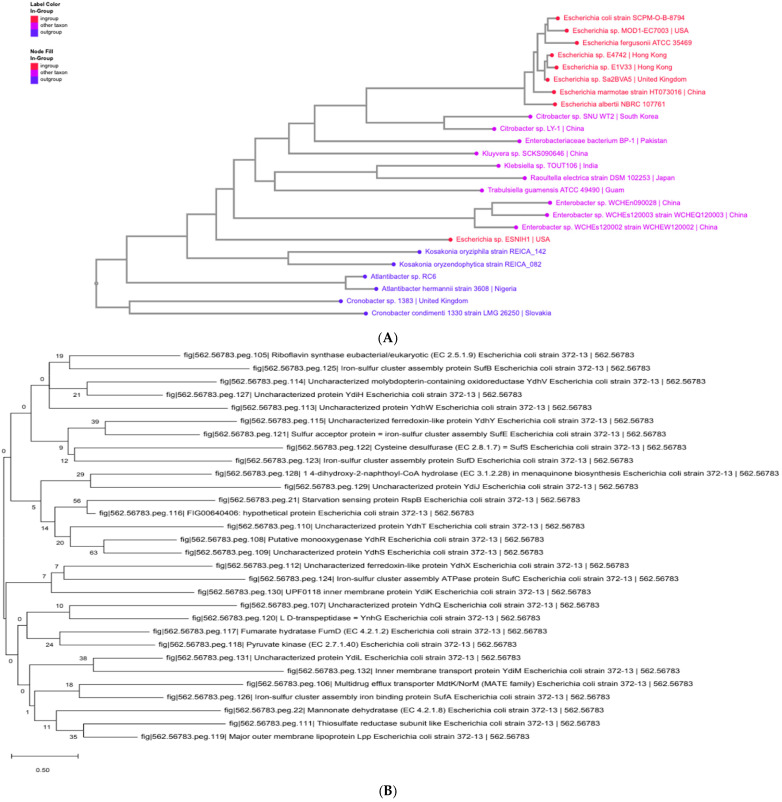
(**A**,**B**) Show a phylogenetic tree comparing *E. coli* clinical isolates with the K-12 reference strain. The phylogenetic relationships among *E. coli* clinical isolates analyzed in this study were aligned against the *E. coli* K-12 MG1655 reference genome. The tree demonstrates that resistant and susceptible isolates form distinguishable clusters, suggesting minor genomic variations linked to antimicrobial resistance traits rather than broad evolutionary divergence. The inclusion of outgroup taxa (*Cronobacter* and *Atlantibacter* species) provided phylogenetic context, confirming that all isolates remained within the Enterobacteriaceae family. These results complement the machine learning classification outcomes by supporting the genomic differentiation patterns underlying phenotypic resistance in *E. coli*.

Moreover, some of the resistant isolates were found to cluster based on geographic origin, with some groups forming closely related branches, which could suggest regional transmission of the resistant lineages. The presence of outgroup taxa, including *Cronobacter* and *Atlantibacter* species, gave some evolutionary context and ensured that the isolates under study were not out of the family of *Enterobacteriaceae*. The results are to be viewed as a descriptive observation since there are no bootstrap support values and formal statistics of clustering in the current analysis. See Figure 7A,B below.

### 3.5. Genomic Biomarker Identification

The genomic biomarker analysis aimed to identify resistance-linked genes and mutations that contribute to the antimicrobial resistance (AMR) phenotypes of *E. coli*. In this regard, two of the most popular AMR databases, namely the Comprehensive Antibiotic Resistance Database (CARD) through the Resistance Gene Identifier (RGI) tool and ResFinder, operated by the Danish Technical University (DTU), were used to analyze the collected genome sequences (BVBRC_genome_feature.fasta). These orthologous tools offered not only curated reference alignments (CARD) but also in silico sequence matching (ResFinder) to identify known AMR determinants, drug classes to which they belong, and resistance mechanisms. The CARD RGI outcome was that there are several *E. coli* genes related to fluoroquinolone, β-lactam, tetracycline, and multidrug resistance. The levels of confidence in the reliability of the test, as well as the strictness and looseness of the test, were reported by CARD to be high in terms of identifying the sequences of genome features. CARD reported that the levels of confidence in the reliability of the test, perfection, strictness, and looseness were high in terms of identifying the sequences of genome features with the known resistance determinants listed in the Antibiotic Resistance Ontology (ARO). Similarly, the ResFinder output identified aminoglycoside-modifying enzymes and β-lactamase variants, consistent with multidrug resistance phenotypes. See Table 2 below, which consists of Genomic Biomarkers of Antimicrobial Resistance Identified from CARD and ResFinder Analysis.

Detected genes correspond to major antibiotic classes, including fluoroquinolones (gyrA, parC), β-lactams (blaTEM, blaCTX-M-15, ampC, blaOXA-1), aminoglycosides (aac(3)-IIa, aac(6′)-Ib-cr), and tetracyclines (tet(A)).

The genomic biomarker analysis provided biological validation of the machine learning predictions by detecting well-defined determinants of antibiotic resistance across multiple antibiotic classes. Several important mutations and resistance genes were consistently observed, underscoring the molecular mechanisms of antimicrobial resistance in *E. coli*.

Among the more significant, we found mutations of the gyrA and parC genes, especially gyrA (S83L, D87N) or parC (S80I) substitutions (R). These mutations are known to alter the target sites of fluoroquinolone antibiotics by modifying DNA gyrase and topoisomerase IV, thereby reducing drug binding and conferring resistance. The identification of these mutations in resistant isolates provides initial support for the predictive performance of the machine learning models and validates their biological relevance. A strong correlation was observed between resistance to beta-lactam drugs and the presence of extended-spectrum beta-lactamases (ESBLs), including blaCTX-M-15, blaOXA-1, and blaTEM. These genes encode enzymes that break down beta-lactam antibiotics, rendering them ineffective. Based on their detection in this study, ESBL-producing *E. coli* has been reported globally as a leading contributor to multidrug resistance in clinical and community settings. Aminoglycoside resistance was associated with the presence of the resistance-modifying enzymes aac(3)-IIa and aac(6′)-Ib-cr, which act to modify the antibiotic molecules, making them less effective chemically. Notably, the AAC (6″)-Ib-cr gene is also associated with low susceptibility to fluoroquinolones, suggesting overlapping resistance mechanisms among drug classes.

Multidrug resistance was linked to efflux pump systems such as acrA, acrB, and mdtK, which actively extrude antibiotics from the bacterial cell. These efflux systems reduce intracellular drug concentrations and confer cross-resistance to structurally diverse antibiotics. The voltage of target-site mutations, enzymatic inactivation, and efflux-mediated resistance mechanisms highlights the complicated and multifactorial nature of antimicrobial resistance as occasioned for *E. coli*. This is essential because these insights complement machine learning outcomes by providing mechanisms for exceptional insight into the traits underlying model predictions. This blending of computation and biological analysis of results rewrites the model, making it potentially applicable to medical science and public health objectives.

## 4. Discussion

In this study, a combined bioinformatics and machine learning approach was used to classify antimicrobial resistance (AMR) phenotypes and identify genomic biomarkers in *E. coli* clinical isolates. Using phenotypic antimicrobial susceptibility testing (AST) data from the Bacterial and Viral Bioinformatics Resource Center (BV-BRC), several machine learning models were developed to predict resistance patterns. These included Random Forest (RF), eXtreme Gradient Boosting (XGBoost), Support Vector Machine (SVM), Logistic Regression (LR), and k-Nearest Neighbors (kNN). The XGBoost model demonstrated superior predictive accuracy (0.86) and ROC-AUC (0.932), showing high generalizability and the ability to classify resistance phenotypes. Although both XGBoost and Random Forest performed well, XGBoost was considered better due to its greater capacity to model nonlinear relationships and feature interactions. The Random Forest model also performed well, with an accuracy of 0.82, and offered interpretability through feature importance, providing biological insights into the key predictors of resistance. Models such as SVMs and logistic regression showed lower accuracy due to their limitations as linear classifiers in high-dimensional biological data and multi-class scenarios. These results suggest that tree-based algorithms, especially ensemble methods, are highly suitable for predicting resistance from phenotypic data.

The dataset was not evenly distributed, with more resistant isolates than susceptible ones (≈68% vs. 32%). This imbalance could have affected the performance of the models, particularly precision and recall, since models tend to favor the majority class. To ensure a fair evaluation, multiple metrics, such as F1-score and ROC-AUC, were considered rather than relying solely on accuracy. This approach provides a more balanced evaluation of model performance under imbalance conditions [21].

However, the relatively moderate precision and recall values (~0.55–0.57) indicate significant trade-offs in classification performance despite the fact that XGBoost and Random Forest showed good performance in terms of accuracy and ROC-AUC. When it comes to predicting antibiotic resistance, these metrics have obvious clinical implications. False negatives, in which resistant isolates are misidentified as susceptible, increase the risk of illness and inappropriate antibiotic use. However, false positives may lead to unnecessary administration of broad-spectrum or last-resort antibiotics, thereby hastening the emergence of antimicrobial resistance [31]. As a result, it is important to consider not only the model’s accuracy but also its misclassification capability and its influence on clinical decision-making. These results help us to understand the significance of optimizing models, not only in their overall performance but also in their reduction in clinically critical errors, especially false positives in high-risk infections.

The genomic biomarkers in this study, such as gyrA, parC, and blaCTX-M-15, are established resistance determinants that have been reported in earlier studies. Here, the findings are not claimed to be new discoveries but rather biological confirmation of the machine learning predictions. The association of observed patterns of resistance with known genetic mechanisms enhances the interpretability of the models and helps to justify their potential to represent biologically meaningful signals. Resistance genes identified in genomics by the Comprehensive Antibiotic Resistance Database (CARD) and ResFinder have been highlighted as important. GyrA mutations at S83L and D87N, along with parC mutations at S80I, were linked to fluoroquinolone resistance, supporting the original research suggesting these genetic mutations were associated with changes in DNA gyrase and topoisomerase IV [32]. Similarly, the observed β-lactamase genes were blaCTX-M-15, blaOXA-1, and blaTEM, which confer resistance to cephalosporins, including third-generation cephalosporins. These enzymes belong to the extended-spectrum β-lactamases (ESBLs), which have been widely detected in *E. coli* isolates from both hospital and community settings [16,33].

Multidrug efflux systems (acrA, mdtK, and emrB) were also determined, and aac(3)-IIa and aac(6′)-Ib-cr were identified as aminoglycoside-modifying enzymes. The co-occurrence of genes for efflux pumps and beta-lactamases suggests the evolution of multidrug resistance through synergistic processes. The efflux pumps also increase cross-resistance to structurally unrelated antibiotics, in addition to lowering intracellular drug concentration, as it is becoming increasingly prevalent in *E. coli* and other Enterobacteriaceae [34]. The presence of efflux-related mechanisms observed in this experiment indicates the genomic plasticity of *E. coli* and its ability to evolve under the pressure of an antibiotic test. This combination of machine learning and genomic validation offers a more reliable framework of AMR prediction versus approaches that depend exclusively on predictive performance, lacking biological context.

The isolates showed greater evolutionary background in the phylogenetic analysis. Resistance clusters were more likely to be observed among resistant isolates, indicating the expansion of some high-risk lineages that carry multiple AMR determinants. This clustering has also been seen in worldwide comparative genomic analysis, where AMR genes often cluster with mobile genetic elements and horizontal gene transfer [35]. This level of genomic plasticity underscores why phylogenetics should be used alongside machine learning predictions to gain a more comprehensive view of the evolutionary processes underlying resistance. But no quantitative clustering measures or bootstrap support were provided, and hence, the presentation of these results must be considered as descriptive.

Together, combining phenotypic, genomic, and phylogenetic data creates a powerful framework for AMR surveillance and prediction. This approach can be used in both clinical and population health labs, with adjustments that enable quick, evidence-based decisions about antimicrobial treatment. Additionally, detecting key biomarkers such as gyrA, blaCTX-M-15, and aac(6)-Ib-cr offers potential molecular targets for developing diagnostic tests and conducting epidemiological surveillance.

In addition to model performance, it is relevant to the practical application of this framework in clinical settings. Because the model uses phenotypic antimicrobial susceptibility testing (AST) data, which is routinely generated in clinical labs, it can be implemented within current workflows without requiring additional genomic sequencing. This is especially helpful in environments where genomic resources are limited. Predictions can also be generated in real time after AST results are obtained, and these results can aid rapid clinical decisions. For example, clinicians can use these predictions to anticipate resistance patterns and adjust antibiotic therapy in advance. In practice, the model can be used as a decision-support tool within antimicrobial stewardship programs, supplementing laboratory outcomes and clinical judgment rather than replacing them.

Although this study achieved its goal of categorizing AMR phenotypes and detecting genomic biomarkers, a larger, more diverse dataset covering various regions of the world and host populations should be used in the future. Predictive accuracy could also be improved by integrating transcriptomic or proteomic layers, thereby allowing the regulatory networks underlying resistance expression to be observed. Further testing of the proposed biomarkers, including experimental validation of the findings through quantitative PCR and functional assays, is also essential to verify their biological significance and diagnostic capabilities. This was a cross-sectional study that lacked temporal or geographic validation, potentially limiting generalizability. The next line of work should test the model with independent dataset on other areas and time frames.

The biological validation provided by integrating genomic biomarker analysis with machine learning predictions is an important layer. The identification of resistance-associated genes such as gyrA, parC, and blaCTX-M-15 indicates that the machine learning models are not only helping achieve high predictive accuracy but are also learning biologically important patterns in the data. This agreement between predictions from computational models and known mechanisms of resistance makes the model more interpretable and distinguishes this work from methods that rely solely on predictive performance without biological context.

Overall, this research showed that machine learning models, particularly XGBoost and Random Forest, can effectively predict antibiotic resistance in *E. coli* using phenotypic data. It also shows that screening genomic biomarkers can provide additional mechanistic insights. This integrated analytical approach enhances understanding of resistance development and will serve as a foundation for future AMR diagnostics, surveillance, and intervention strategies that can be replicated.

## 5. Conclusions

This research demonstrated that combining phenotypic information and genomic data with machine learning methods could increase predictive capacity for antimicrobial resistance (AMR) in *E. coli.* The XGBoost and Random Forest models demonstrated the best predictive performance among the models considered, suggesting the usefulness of ensemble approaches for handling complex biological data. Genomic analysis also identified the major determinants of resistance, specifically gyrA, which is particularly significant in combination with parC and qnrS, linking well-established mechanisms of fluoroquinolone resistance.

Besides assessing the performance of the evaluation model, the results show that combining machine learning algorithms based on phylogenetic and biomarker analyses provides a more complete view of resistance dynamics. The hybrid approach balances predictive accuracy and biological interpretability, which is essential for making the method useful in a clinical setting.

Future studies aim to include explainable AI models, promoting transparency and building confidence in automated AMR detection. Additionally, efforts are underway to assess the framework across larger, geographically diverse *E. coli* datasets and to expand its use to other priority pathogens. Overall, this research advances data-driven AMR surveillance and takes a scalable step toward transparent, AI-supported diagnostics in microbial genomics.

This study demonstrates that integrating phenotypic and genomic data with machine learning can improve the accuracy of antimicrobial resistance prediction in *E. coli*. Expanding the emerging field of data-driven antimicrobial surveillance provides a robust framework for more accurate diagnostics and evidence-based antibiotic management, strengthening global AMR mitigation efforts.

## Figures and Tables

**Table 1:** Performance of Machine Learning Model on AMR classification.

MODEL	ACCURACY	ROC-AUC	Precision	Recall	F1-Score
Random Forest	0.82	0.890	0.56	0.55	0.56
XGBoost	0.86	0.932	0.57	0.55	0.56
SVM	0.67	0.540	0.56	0.34	0.27
Logistic Regression	0.76	0.637	0.40	0.37	0.35
KNN	0.78	0.828	0.52	0.51	0.52

**Table 2:** Genomic Biomarkers of Antimicrobial Resistance Identified from CARD and ResFinder Analysis.

Gene/Mutation	Antibiotics Class	Phenotypic Resistance/Associated Drugs	Resistance Mechanism	Confidence/Source
gyrA (S83L, D87N)	Fluoroquinolones	Ciprofloxacin, Levofloxacin	Target alteration (DNA gyrase mutation)	Perfect, CARD
parC (S80I)	Fluoroquinolones	Ciprofloxacin	Target alteration (Topoisomerase IV mutation)	Perfect, CARD
aac(3)-IIa	Aminoglycosides	Gentamicin, Tobramycin	N(3)-acetyltransferase enzyme	28636609, ResFinder
aac(6′)-Ib-cr	Aminoglycosides/Fluoroquinolones	Tobramycin, Amikacin, Ciprofloxacin	N(6′)-acetyltransferase (fluoroquinolone acetylation)	DQ303918, ResFinder
blaCTX-M-15	β-lactams/Cephalosporins	Amoxicillin, Cefotaxime, Cefepime, Ceftriaxone	Extended-spectrum β-lactamase (Class A)	11470367, ResFinder
blaOXA-1	β-lactams	Ampicillin, Amoxicillin-clavulanate, Piperacillin	Class D OXA-type β-lactamase	10898672, ResFinder
blaTEM, ampC	β-lactams/Cephalosporins	Ampicillin, Cefazolin	Antibiotic inactivation (β-lactamase)	Strict, CARD
acrA, acrB, acrE, acrF	Multidrug/Quinolones	Multiple drug substrates	Efflux pump complex (RND family)	Strict, CARD
mdtK, mdtH, mdtM, mdtG, mdtN	Multidrug/Macrolides	Erythromycin, Azithromycin	Efflux transporters and regulators	Strict, CARD
emrB, emrR, emrY	Macrolides/Phenicols	Chloramphenicol, Erythromycin	Multidrug efflux and regulatory proteins	Strict, CARD
catB3	Phenicols	Chloramphenicol	O-acetyltransferase enzyme (drug inactivation)	1662753/7793874, ResFinder
tet(A), tetR	Tetracyclines	Tetracycline, Doxycycline	MFS efflux pump system	12654659, ResFinder
msbA, tolC	Disinfectant/Multidrug	Broad substrate range	Membrane transport and antibiotic efflux	Loose, CARD
vanG, vanD	Glycopeptides	Vancomycin	Target alteration (cell wall modification)	Loose, CARD

## Data Availability

All processed data and analysis scripts will be made publicly available via GitHub upon publication. https://github.com/Sarah4God/Antimicrobial-Resistance-Analysis (accessed on 20 April 2026); https://github.com/Sarah4God/Antimicrobial-Resistance-Analysis/tree/main (accessed on 20 April 2026).

## Abbreviations

The following abbreviations are used in this manuscript:

AMR	Antimicrobial Resistance
*E. coli*	*Escherichia coli*
BV-BRC	Bacterial and Viral Bioinformatics Resource Center
CARD	Comprehensive Antibiotic Resistance Database
